# Estrogen preserves split renal function in a chronic complete unilateral ureteral obstruction animal model

**DOI:** 10.3892/etm.2014.1663

**Published:** 2014-04-03

**Authors:** SHANHUA MAO, HUA XU, LUJIA ZOU, GANG XU, ZHONG WU, QIANG DING, HAOWEN JIANG

**Affiliations:** Department of Urology, Huashan Hospital, Fudan University, Shanghai 200040, P.R. China

**Keywords:** estrogen, split renal function, glomerular filtration rate, ureteral obstruction, rat

## Abstract

Estrogen may help to preserve renal function in chronic kidney disease. This study examined whether estrogen administration or deprivation affected the split renal function in rats subjected to chronic unilateral ureteral obstruction (UUO). Fifteen adult female Sprague-Dawley rats were randomly divided into three groups. Low- and high-estrogen groups were modeled by female castration or estrogen intraperitoneal injection, respectively, and the rats in the normal-estrogen group were untreated. Intermittent split renal function [glomerular filtration rate (GFR)] examination was performed on rats on days 2, 6 and 16 after UUO surgery via single-photon emission computed tomography (SPECT/CT). Routine hematoxylin and eosin (H&E) staining, immunohistochemistry, pathology examination and electron microscopy were performed to compare the histological differences. Low-, normal- and high-estrogen groups were successfully established (P<0.001). In the acute stage, the GFR of the contralateral healthy kidney showed a greater compensatory rise in the normal- and high-estrogen groups than in the low-estrogen group (P<0.05). In the chronic stage, the GFR of the obstructed kidney continued to decrease with the GFR of the high-estrogen group being significantly better preserved than that of the low-estrogen group (P<0.05). The GFR of the contralateral kidney compensated to the greatest extent in the high-estrogen group (P=0.01), and the total GFR was significantly superior (P<0.05). Routine H&E examination showed significant histological changes following surgery. The low-estrogen group had significant renal interstitial fibrosis compared with the normal- and high-estrogen groups (P<0.05), as observed by immunohistochemical (IHC) examination of transforming growth factor-β (TGF-β) and α-smooth muscle actin (α-SMA). Electron-microscopic (EM) examination also differentiated between groups. In conclusion, estrogen administration and deprivation significantly affected renal function. Estrogen may preserve the split renal function (GFR) in rats with chronic UUO.

## Introduction

The annual incidence of upper urinary tract calculi has steadily increased in Asia and this trend is likely to continue in the near future (1,2). The calculi may lead to hydronephrosis, a type of renal atrophy that may result in renal insufficiency or a unilateral non-functioning kidney (NFK) (3). In our previous study, a gender difference in the development of NFK was observed among patients with urolithiasis (4). Sex hormones may contribute to this phenomenon.

Estrogen has been implicated in the pathophysiology of chronic kidney disease and has been shown to provide a protective effect against chronic renal damage (5–7). The protective effects likely act through the renin-angiotensin system (8–10), nitric oxide pathway (11,12), extracellular matrix metabolic pathway, inflammatory response pathway (13–17), lipid metabolic pathway and numerous other pathways (18–20).

However, few groups have reported whether estrogen is able to preserve the renal function in unilateral ureteral obstruction (UUO). This study aimed to test the hypothesis that estrogen administration is able to preserve the split renal function in UUO and that estrogen deficit has a harmful effect.

## Material and methods

### Experimental model

Experiments were performed in 15 female Sprague-Dawley (SD) rats [supplied by the Department of Laboratory Animal Science, Fudan University, Shanghai, China; Certificate No. 2009001901383, SCXK (Shanghai) 2009-0019] aged 6–8 weeks and weighing 200±10 g. The study was carried out in strict accordance with the Guide for the Care and Use of Laboratory Animals (8th edition, 2011). The protocol was approved by the Animal Welfare and Ethics Committee, Fudan University. Female castration, UUO creation and single-photon emission computed tomography (SPECT/CT) were performed under anesthesia of sodium pentobarbital (50 mg/kg, intraperitoneally).

For the low-estrogen model, female castration (ovariectomy, OVX) was performed with the removal of both ovaries and a small section of uterus through two small waist incisions. To create the high-estrogen model, estrogen with a vehicle of tea oil injection was intraperitoneally injected (10 μg/rat) on alternate days. To create the normal-estrogen model, the rats were left untreated. After 3 weeks, the blood was withdrawn from the caudal vein for the determination of serum estrogen concentrations.

For UUO creation, a low midline abdominal incision was made under anesthesia. After the ureter was mobilized and isolated with minimal dissection, it was ligated with two 4-zero silk sutures (Johnson-Johnson, Shanghai, China) at the ureterovesical junction. The wound was sutured using 2% lidocaine solution as an anaesthetic and hydropathically compressed (Shanghai Zhaohui Pharmaceutical Co., Ltd., Shanghai, China) to relieve pain.

### Study design

The rats were randomly divided into three groups: high- (5 rats, Estrogen administration + UUO), normal- (5 rats, only UUO) and low- (5 rats, OVX + UUO) estrogen groups. Radioimmunoassay of estrogen was performed to confirm the different estrogen levels. UUO was performed followed by 3 weeks of estrogen modeling. On days 2, 6 and 16 after surgery, split renal function [glomerular filtration rate (GFR)] was measured by SPECT/CT with 99mTc-labelled diethylene triamine pentaacetate (DTPA). A preliminary experiment showed marked nephrosis and cystic changes of the obstructed kidney on day 16; therefore, pre-surgery and post-surgery (day 17) serum creatinine levels were also measured. Routine immunohistochemistry (IHC), pathology and electron-microscopic (EM) examinations were performed to compare histological differences. The rats were sacrificed with an overdose of anesthetic (sodium pentobarbital, 100 mg/Kg, intraperitoneally).

### Evaluation

Evaluation included split renal function (GFR), serum creatinine, pathology and EM examinations for all three groups.

For the evaluation of GFR, the rat was fastened to the scanning table in the supine position under anesthesia. Bolus injection of 99mTc-DTPA (30 μCi/100 g) was carried out intravenously and a standard renal scan was performed. In order to avoid random errors, the results were calculated three times by three professional doctors of nuclear medicine and then averaged.

For serum creatinine evaluation, a blood sample was withdrawn from the caudal vein or inferior vena cava and subjected to biochemical analysis.

For pathology and EM examinations, specimens of both kidneys were fixed by glutaraldehyde and formalin. EM examination was performed on a Philips CM120 transmission electron microscope (Philips, Amsterdam, The Netherlands). Hematoxylin and eosin (H&E) staining, and IHC staining of TGF-β and α-SMA were performed on paraffin-embedded tissue blocks. The H&E-stained slides were reviewed by the same pathologist. The positive staining area percentages of TGF-β and α-SMA slides were measured using Image-Pro Plus (IPP) V 6.0 software (Media Cybernetics, Rockville, MD, USA) with an average of five high magnification fields for each slide. The slides were visualized under the same circumstances at the same time.

### Statistical analysis

SPSS software (version 19.0; IBM, Armonk, NY, USA) was used for statistical analysis. The groups of data were normally distributed with P>0.05. The Student’s t-test and analysis of variance test were used to compare the data of three groups for categorical variables. P<0.05 was considered to indicate a statistically significant result.

## Results

A total of 15 female SD rats were included in the present study with five rats in each group. Three rats died of accidental anesthesia overdose. No other complications were observed

### Changes in serum estrogen

Blood samples was obtained after 3 weeks of estrogen modeling by medication or surgery. The concentration of estrogen was 89.01±11.19 pg/ml for the low-estrogen group, 135.97±26.23 pg/ml for the normal-estrogen group and 209.68±13.86 pg/ml for the high-estrogen group (P<0.001). Thus, the various estrogen animal models were established successfully.

Prior to modeling, the rats appeared identical with the same weight, body morphology and hair color pattern. After 3 weeks of modeling, the rats in the low-estrogen group appeared fatter and had significantly less lustrous hair when compared with the rats in the other groups.

### Changes in split glomerular filtration rate

**Figure 1:**
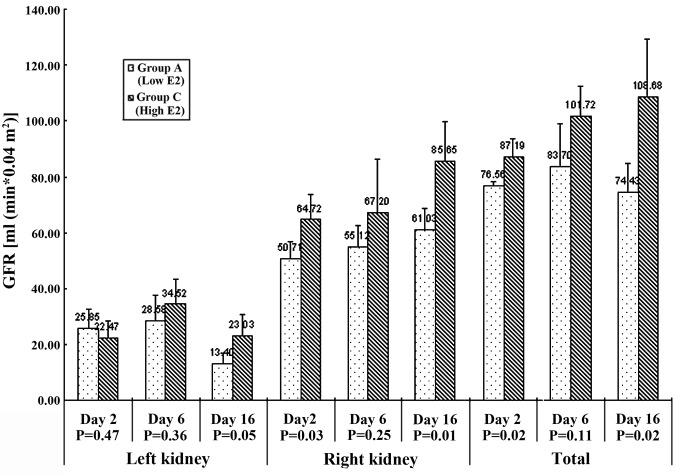
GFR of low- and high-estrogen level groups (group A, low estrogen; group C, high estrogen). The GFR of the obstructed kidney significantly declined while that of the high-estrogen level group was better preserved. The contralateral kidney also compensated better for the GFR of the obstructed kidney in the high-estrogen level group. GFR, glomerular filtration rate.

The GFRs of rats were compared prior to surgery and on days 2, 6 and 16 after surgery (Table I, Fig. 1). On day 0, there was no significant difference in bilateral GFR among the three groups. In the acute (early) stage (day 2), the GFR of the obstructed kidney was significantly decreased (P<0.05) in the three groups while the GFR of the contralateral healthy kidney showed a greater compensatory rise in the normal- (67.70±1.15 ml/min/0.04 m^2^) and high-estrogen (64.72±9.25 ml/min/0.04 m^2^) groups than in the low-estrogen (50.71±6.25 ml/min/0.04 m^2^) group (P<0.05). The same trend was observed for the total GFR.

In the medium stage (day 6), the GFR of the obstructed kidney remained significantly lower than that of the contralateral side (P<0.05). For the healthy side, the three groups reached the same compensatory level with no significant difference (P=0.437).

In the chronic (late) stage (day 16), the GFR of the obstructed kidney continued to decrease with the high-estrogen group (23.03±7.88 ml/min/0.04 m^2^) being significantly better preserved than the low-estrogen group (13.40±3.64 ml/min/0.04 m^2^) (P=0.05). The GFR of the contralateral healthy kidney compensated more in the high-estrogen group (85.65±14.13 ml/min/0.04 m^2^) than in the low- and medium-estrogen groups (61.03±7.45 and 55.20±3.37 ml/min/0.04 m^2^, respectively) (P=0.01). The same trend was observed for change of total GFR (P<0.05).

### Changes in serum creatinine

Pre- and post-surgery serum creatinine levels showed no differences among the groups (P>0.05) while significant increases existed within each group prior to and following surgery (P<0.01; Table I).

### Changes in pathology and EM visualization

**Figure 2:**
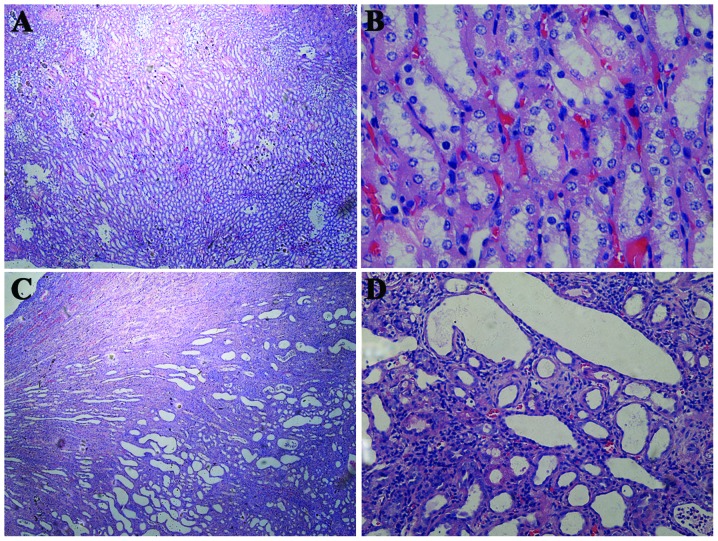
(A and B) Hematoxylin and eosin staining of healthy kidney (magnification, ×5 and ×100, respectively). (C and D) Hematoxylin and eosin staining of obstructed kidney. Conspicuous tubulointerstitial injury characterized by tubular dilatation and atrophy, interstitial inflammation and a marked interstitial fibrosis were observed (magnification, ×5 and ×40, respectively).

H&E staining revealed no significant difference in the damage among the three groups. However, on day 17 (chronic stage), compared with the healthy kidneys, the obstructed kidneys developed a conspicuous tubulointerstitial injury characterized by tubular dilatation and atrophy, interstitial inflammation and a marked interstitial fibrosis (Fig. 2).

**Figure 3:**
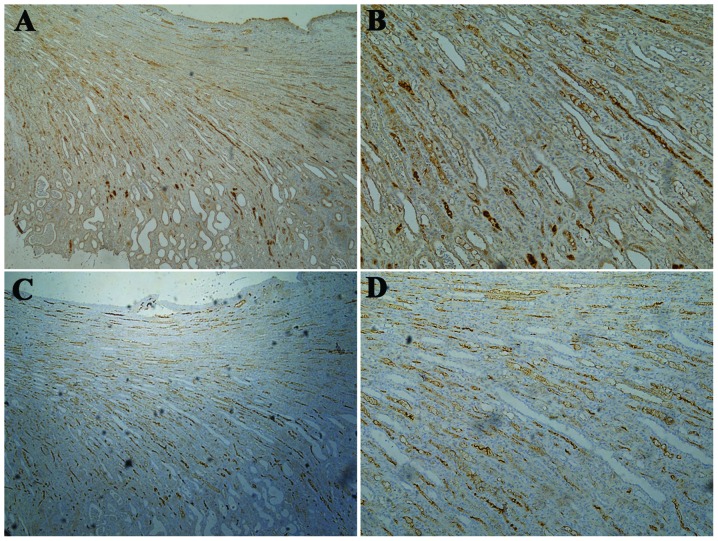
(A and B) IHC staining of TGF-β. TGF-β expression was observed in the cytoplasm of the tubular epithelial cells. (C and D) IHC staining of α-SMA. α-SMA expression was also observed in renal interstitium (magnification, ×5). IHC, immunohistochemical; TGF-β, transforming growth factor-β; α-SMA, α-smooth muscle actin.

During IHC examination, significant renal fibrosis was analyzed using IPP V 6.0 software to measure the positive staining area percentage of TGF-β and α-SMA (Fig. 3, Table II). TGF-β is a protein that controls proliferation, cellular differentiation and other functions in the majority of cells, and is an important target for renal interstitial fibrosis. With the reduction in estrogen concentration, enhanced TGF-β expression was observed in the cytoplasm of the tubular epithelial cells in the present study. The low-estrogen group had a significantly higher expression of TGF-β than the other two groups (P=0.05 vs. the normal-estrogen group; P=0.03 vs. the high-estrogen group). α-SMA is a protein that is involved in cell motility, structure and integrity. α-SMA is a major constituent of the contractile apparatus and is commonly used as a marker of myofibroblast formation. With the reduction in estrogen concentration, enhanced α-SMA expression was also observed in the renal interstitium in the present study. The low-estrogen group had a significantly higher expression of α-SMA than the high-estrogen group (P=0.008).

**Figure 4:**
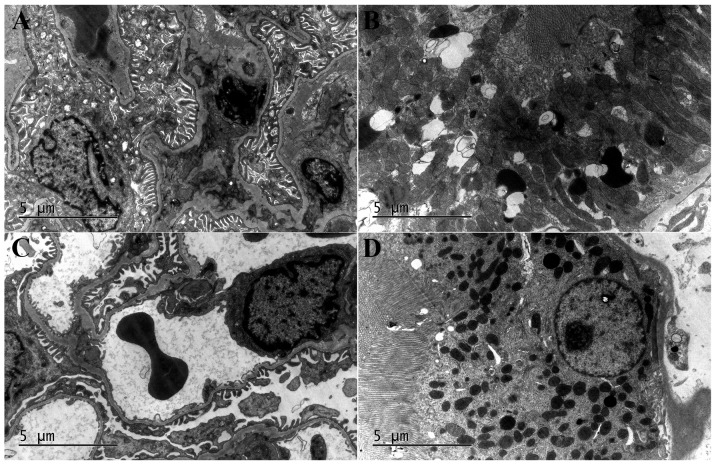
(A and B) EM examination of healthy kidney. Normal capillary endothelial cells, basement membrane, epithelial cells and foot processes were observed. (C and D) EM examination of obstructed kidney. Changes typical of hydronephrosis were observed, including increased numbers of epithelial cells, swelling, shedding and vacuolization change of mitochondrial of epithelial cells in the renal glomerulus area and autophagic vacuoles, vacuolization and lipofuscin in the proximal tubule area. EM, electron-microscopic.

Under EM examination, no significant difference in damage was identified among the three groups. Normal capillary endothelial cells, basement membranes, epithelial cells and foot processes in healthy kidneys were observed. In addition, no obvious abnormalities in the capillary lumen with erythrocytes within the renal glomerulus area and normal mitochondria, proximal tubule epithelial cells and vertically arranged microvilli in the proximal tubule area were observed in the healthy kidneys. For the obstructed kidneys, changes typical of hydronephrosis were observed, including increased numbers of epithelial cells, swelling, shedding and vacuole formation in the mitochondria of epithelial cells in the renal glomerulus area, and autophagic vacuoles, vacuolization and lipofuscin with no evident abnormalities in the mitochondria and microvilli in the proximal tubule area (Fig. 4).

## Discussion

Urinary tract stones may occasionally be solved without any treatment, but may also lead to heavy hydronephrosis and NFK. We previously conducted a survey which found that the prevalence of NFK remained constant regardless of the increased use of endoscopic techniques and screening, with females, in particular elderly women, being more likely to develop NFK (4). Therefore, the gender difference and the effect of estrogen was investigated using a UUO model in the present study.

The treatment of obstructive nephropathy is by relief of obstruction. However, while patients are undergoing conservative medical treatment of small ureteral stones, waiting for extracorporeal shock wave lithotripsy (SWL) or ureteroscopy treatment, or even after SWL or ureteroscopy treatment, the corresponding ureter remains obstructed during passage. For certain patients, such obstruction may remain for several weeks. Therefore, preservation of the renal function under such conditions is invaluable. In addition, certain studies have observed that the harmful effect of obstructive nephropathy may continue even after relief of the obstruction. The recovery of renal function is a long-term process (21). Thus, the identification of a medicine that is able to augment the progression of renal function is likely to be valuable for clinical application.

The present study is a controlled experiment to evaluate the role of estrogen in the progression of renal function loss during a phase of chronic complete ureteral obstruction from the perspective of morphological and functional study. To the best of our knowledge, these objectives have not been achieved in published urological studies.

From the point of functional study, estrogen contributed to a renoprotective effect during UUO. The high-estrogen group exhibited a better preserved split renal function (GFR) for the obstructed kidney and superior compensatory effect for the contralateral kidney, particularly during the chronic stage.

The creatinine level does not differentiate between impaired split renal function and reflects only the total renal function. The rise in creatinine level did not differ among groups due to the compensatory effect of the healthy kidney. Thus, SPECT/CT is better than creatinine level monitoring for evaluating the split renal function more precisely.

From the point of morphological study, estrogen also contributes to the renoprotective effect of interstitial fibrosis during UUO. Significant hydronephrosis changes and inflammatory infiltration were observed in the obstructed kidney by H&E staining and EM examination. In addition, IHC staining of TGF-β and α-SMA was able to differentiate between different estrogen level groups. TGF-β and α-SMA are the typical markers of renal interstitial fibrosis. TGF-β controls cell proliferation and is one of the most important signs of renal fibrosis involved in Smad signaling pathways (22). α-SMA is a marker of myofibroblast formation, which plays a crucial role in the development and progression of renal tubulointerstitial fibrosis (23). In the present study, the low-estrogen group demonstrated significantly deeper staining of these two markers, which indicated that severe renal interstitial fibrosis had occurred and a high estrogen level was able to downregulate its progression.

As observed in a previous study (4), the majority of female NFK patients with urolithiasis were postmenopausal (mean, 53.4±13.3 years old). This may be due to the sharp decline in estrogen levels following menopause causing the loss of a renoprotective effect of renal function for female patients with urolithiasis and leading to NFK. The present study has demonstrated, for the first time, that estrogen is able to protect renal functions in acute and chronic UUO.

Estrogens are a group of compounds named for their importance in the estrous cycle of humans and other animals. They are the primary female sex hormones. Estrogens, in females, are produced primarily by the ovaries and, during pregnancy, the placenta. Estrogens are also produced in smaller amounts by other tissues such as the liver, adrenal glands and breasts. For adolescent girls, estrogen is able to promote formation of female secondary gender characteristics, produce libido (24), regulate the fluid balance (25) and cause calcium deposition in the bones.

Previous study has shown that estrogen is able to alleviate the progression of chronic kidney disease (5). Therefore, the high prevalence of NFK in older women with urolithiasis in our previous study may be due to the sharp decline of estrogen levels (4). Ji *et al* observed that estrogen influenced the severity of injury in a renal wrap-induced renal injury animal model; estrogen treatment protected against glomerular and tubular damage (5). Fung *et al* also performed a cross-sectional and 10-year prospective study of postmenopausal estrogen therapy and observed improved blood pressure and renal function (GFR) among continuous estrogen users (6).

In addition, estrogen is involved in multiple pathways in the progression of obstructive nephropathy. The arachidonic acid metabolic, renin-angiotensin, nitric oxide, aquaporin and extracellular matrix pathways, together with transport disorders of sodium and potassium, the failure of urine acidification due to hydrogen ion transport disorders, and secretion disorders of a variety of peptides and proteins are involved in the development of obstructive nephropathy (26,27). Estrogen is involved in many of these pathways. For renin-angiotensin pathways, Baiardi *et al* observed that estrogen was able to upregulate the angiotensin II receptor (AT2R) to protect renal function (9). Oelkers (28) and Gallagher *et al* (29) also found that estrogen was able to upregulate angiotensinogen and the AT2R, and downregulate renin, angiotensin-converting enzyme and angiotensin II, which also protected renal function. For nitric oxide pathways, Thompson and Khalil found that estrogen activates endothelium-dependent vascular relaxation pathways, including NO-cGMP and prostacyclin-cAMP pathways, which has potential beneficial vascular effects (11). Sandberg also analyzed nitric oxide synthesis (NOS) disorders in a renal wrap model of hypertension in rats and observed that estrogen regulates NOS and NO to preserve renal function (12). For extracellular matrix pathways, Karl *et al* found that estrogen *in vitro* prevented TGF-β1 stimulation of a Smad-responsive reporter construct and increased MMP-2 expression and activity that alleviated renal interstitial fibrosis (30). This study also demonstrated that estrogen downregulated TGF-β. Guccione *et al* found that estrogen was able to upregulate the MAPK cascade, which in turn stimulated the synthesis of AP-2 protein. The resultant increased AP-2/DNA binding activity leads to increased synthesis of MMP-2 and increased metalloproteinase activity, which may contribute to the protective effect of female gender on renal disease progression (31). Zdunek *et al* (32) found that the ability of estrogen to reverse TGF-β1-stimulated type IV collagen synthesis was mediated by the downregulation of CK2 activity and ultimately collagen IV protein synthesis was reduced. Neugarten *et al* (33) obtained similar results. In addition, estrogen is able to inhibit podocyte injury (18) and mesangial apoptosis (20), stimulate vascular endothelial growth factor (VEGF) expression to maintain the healthy intrarenal vasculature (19) and preserve renal function. Estrogen is involved in pathways that may mediate the progression of obstructive nephropathy.

Women <60 years old with low bone density, flushes, sweats, vaginal dryness, loss of libido and climacteric depression are treated with estrogen (hormone replacement therapy, HRT) by gynecologists and the majority of general practitioners. However, the popular use of estrogen has been reduced by the 2002 Women’s Health Initiative study due to adverse effects, including increased risk of breast cancer, endometrial cancer, thromboembolic disease and stroke being reported (34). A previous study suggested that HRT is more likely to be a tumor promoter than a *de novo*-inducer (35). So, whether to use HRT or not remains unclear. At present, the recommendations state categorically that the safety of HRT largely depends on age, adding that healthy women <60 years old should not be unduly concerned about the safety profile of HRT (36). Therefore, the benefits of HRT given for a clear indication are many and the risks are few. Further study is required to investigate the merit and demerit of the use of estrogen (HRT).

The limitations of the present study include a small sample size and the use of a complete UUO animal model rather than a partial UUO model. However, laboratory, functional and morphological examinations were performed to obtain further information. Three professional nuclear medicine doctors calculated the GFR and five high magnification fields were obtained for each IHC slide to calculate the expression of TGF-β and α-SMA to minimize bias. A partial UUO model is relatively difficult to establish with a consistent degree of obstruction that affects the renal function. Therefore a complete UUO was used to maintain consistency of the experimental conditions. Future studies to design a partial UUO model and further investigate the mechanism of estrogen are required.

In conclusion, estrogen administration preserved the renal function of obstructive kidney in a unilateral ureteral obstruction animal model and enhanced the compensatory effect of the contralateral kidney. SPECT/CT examination (GFR) is an effective method of measuring split renal function.

## Figures and Tables

**Table I:** GFR and serum creatinine of three estrogen level groups [group A, low estrogen (n=4); group B, normal estrogen (n=3); group C, high estrogen (n=5)].

	Group			P-value			
		
Variable	A	B	C	ANOVA	A vs. B	B vs. C	A vs. C
Estrogen (pg/ml)	89.01±11.19	135.97±26.23	209.68±13.86	0.000	0.022	0.002	0.000
Serum creatinine (μmol/m)							
Day 0	30.75±0.50	42.33±15.31	36.20±3.83	0.196	0.178	0.405	0.027
Day 17	52.75±1.89	51.00±13.45	59.40±13.16	0.523	0.801	0.435	0.356
P-value	0.000	0.502	0.015				
GFR (ml/min/0.04 m^2^) (days after surgery)							
Left kidney							
Day 0	51.19±2.23	50.37±2.54	51.10±2.92	0.908	0.670	0.733	0.964
Day 2	25.85±6.95	33.64±6.24	22.47±6.01	0.107	0.184	0.065	0.470
Day 6	28.58±8.95	42.56±17.89	34.52±9.05	0.332	0.309	0.528	0.360
Day 16	13.40±3.64	22.69±7.76	23.03±7.88	0.124	0.162	0.955	0.052
Right kidney							
Day 0	50.71±2.73	50.64±4.52	51.66±3.80	0.901	0.980	0.741	0.687
Day 2	50.71±6.25	67.70±1.15	64.72±9.25	0.021	0.006	0.514	0.031
Day 6	55.12±7.79	58.71±6.37	67.20±19.07	0.437	0.533	0.400	0.249
Day 16	61.03±7.45	55.20±3.37	85.65±14.13	0.005	0.232	0.012	0.014
Total							
Day 0	101.90±3.85	101.01±6.25	102.76±5.77	0.903	0.824	0.699	0.804
Day 2	76.56±11.68	101.34±6.01	87.19±6.41	0.001	0.015	0.029	0.018
Day 6	83.70±15.60	101.27±19.87	101.72±10.82	0.201	0.278	0.974	0.105
Day 16	74.43±10.50	77.90±11.02	108.68±20.86	0.021	0.694	0.034	0.021

GFR, glomerular filtration rate.

**Table II:** Semi-quantitative analysis (Image-Pro Plus V6.0) for IHC staining of the three estrogen level groups [group A, low estrogen (n=4); group B, normal estrogen (n=3); group C, high estrogen (n=5)].

	Positive percentage			P-value			
		
Protein	Group A	Group B	Group C	ANOVA	A vs. B	B vs. C	A vs. C
TGF-β	58.83±9.47	40.36±13.04	42.93±10.81	0.05	0.05	0.76	0.03
α-SMA	38.97±10.19	32.24±10.75	19.09±9.37	0.02	0.41	0.10	0.01

IHC, immunohistochemical; TGF-β, transforming growth factor-β; α-SMA, α-smooth muscle actin.
